# Comparative Assessment of the Knowledge, Awareness, and Practices Regarding an Artificial Intelligence Tool (Chat Generative Pre-trained Transformer or ChatGPT) Among Dental Undergraduate and Postgraduate Students and the Teaching Faculty

**DOI:** 10.7759/cureus.79031

**Published:** 2025-02-15

**Authors:** Sameer A Zope, Sayali S Dongare, Girish Suragimath, Siddhartha Varma, Apurva V Kale

**Affiliations:** 1 Department of Periodontology, School of Dental Sciences, Krishna Vishwa Vidyapeeth (Deemed To Be University), Karad, IND

**Keywords:** artificial intelligence, chatgpt, dental education, dentistry, neural network

## Abstract

Introduction

Artificial Intelligence (AI) is transforming dentistry by enhancing diagnostics, treatment planning, and education. Chat Generative Pre-trained Transformer (ChatGPT), a conversational AI, offers the potential for improving communication, academic learning, and clinical decision-making. However, challenges like data privacy, ethical implications, and limited validation remain. This study evaluated the knowledge, awareness, and practices regarding ChatGPT among dental students and the teaching faculty in Maharashtra, India.

Methods

A cross-sectional study was conducted among 384 participants: 161 undergraduates, 138 postgraduates, and 85 members of the teaching faculty. A pre-validated, closed-ended questionnaire with 22 questions assessed their knowledge, awareness, and practices regarding ChatGPT. Data were collected via Google Forms and analyzed using Pearson’s chi-squared test, with p<0.05 considered significant.

Results

A total of 384 participants, including undergraduates (n=161), postgraduates (n=138), and members of the teaching faculty (n=85), were surveyed about their knowledge, awareness, and use of ChatGPT in dentistry. The majority (87.2%) recognized ChatGPT as an AI-based neural network and were aware of its role in guiding patients and assisting in clinical decision-making. Postgraduates showed the highest knowledge and acceptance. Concerns about data privacy were prominent, especially among the teaching faculty (91.8%). Postgraduates frequently used ChatGPT for additional dental information and as a knowledge repository. However, faculty members were less likely to use ChatGPT for presentations, with significant differences observed across groups (P<0.05).

Conclusion

ChatGPT is gaining recognition for its diverse applications in dentistry, with the teaching faculty demonstrating increased awareness and postgraduate students demonstrating better knowledge and greater practical use. Integrating AI into dental education and developing targeted training programs can enhance its role in advancing education, research, and patient care.

## Introduction

Artificial Intelligence (AI) is transforming the field of dentistry, bringing unprecedented precision, efficiency, and patient-centered care to every aspect of practice. From diagnostic imaging to personalized treatment planning and patient management, AI-driven tools enable dental professionals to elevate clinical outcomes and streamline operations [1]. The ability of AI to process vast amounts of data with speed and accuracy offers a new level of insight, empowering dentists to detect issues earlier, craft highly personalized treatment plans, and anticipate patient needs [2]. This technology is reshaping the dental experience, making care more accessible, effective, and affordable.

Tracing its roots back to the 1950s, AI has evolved from theoretical algorithms to advanced applications in healthcare, with dentistry leading the way in adopting AI for both clinical and educational purposes [3]. Today, AI technologies such as machine learning, computer vision, and natural language processing support precise diagnostics, enhance patient communication, and facilitate remote learning for dental students. Chat Generative Pre-trained Transformer (ChatGPT), a conversational AI model, promises to strengthen communication, education, and clinical decision-making in dentistry [4]. The integration of ChatGPT in dentistry signifies a significant leap forward in the fusion of AI and healthcare. Its ability to understand natural language, provide accurate information, and assist patients and practitioners can revolutionize dental care delivery, making it more accessible, efficient, and patient-centered [5]. In radiology, convolutional neural networks (CNNs) aid in detecting dental caries, apical lesions, and maxillary sinusitis with great accuracy [6]. Focusing on periapical radiographs, CNN achieved 81.0% and 76.7% accuracy in diagnosing periodontally compromised premolars and molars [7]. AI-powered tools also enhance orthodontics by automating cephalometric analysis and improving treatment precision. In prosthodontics, AI streamlines workflows, such as designing dentures and optimizing material selection [8]. Furthermore, AI supports endodontics by identifying root fractures and analysing canal anatomy [9]. While promising, these applications require further validation to ensure reliability and ethical adherence in clinical settings. However, while AI’s applications in diagnostics, treatment planning, and workflow optimization have been widely explored, there is limited research on the awareness and practical use of AI tools like ChatGPT among dental professionals.

Hence, this study aims to assess the knowledge and awareness of ChatGPT among dental undergraduate students, postgraduate students, and faculty members. Understanding their familiarity with and perceptions of AI in dental education and practice can help identify gaps and inform strategies for integrating AI-based tools into the curriculum. By evaluating levels of awareness and utilization, this study seeks to provide insights into how AI adoption can be optimized in dental academia and practice.

## Materials and methods

Study design

This cross-sectional questionnaire-based study was conducted among 384 participants from various dental colleges across India, comprising 161 undergraduate students, 138 postgraduate students, and 85 members of the teaching faculty. Ethical clearance was obtained from the ethics review committee of Krishna Vishwa Vidyapeeth before commencing the study. A participant information sheet was provided, and informed consent was obtained from all participants before enrollment. Data on prior AI exposure and experience were collected to better understand the background of the participants. Participants were asked whether they had previously used AI-based tools, including ChatGPT, and whether they had received any formal education or training in AI applications in dentistry. The inclusion criteria consisted of postgraduate and undergraduate students, as well as teaching faculty who voluntarily consented to participate. Those unwilling to participate or who did not complete the questionnaire were excluded from the study. Anonymous responses were collected to minimize information bias and encourage honest feedback.

Sample size

The sample size was estimated to be approximately 376 participants, considering an alpha error of 5% and a study power of 80%, using the formula n = (E² × p × (1 - p)) / Z². A total of 384 complete and valid responses were received, meeting the required sample size for statistical analysis.

Ethical statement 

The ethical clearance was obtained from the Ethics Review Committee of Krishna Vishwa Vidyapeeth (ethical clearance protocol number: 409/2023-2024), before commencing the study.

Pre-validation and pre-testing of the questionnaire

The questionnaire was pre-tested and pre-validated by the experts from the Krishna Vishwa Vidyapeeth protocol committee. A pilot study among 30 participants was also conducted to assess the reliability and validity of the questionnaire. A specially designed, closed-ended Google Form questionnaire (Google LLC, Mountain View, CA, USA) was created, consisting of 22 questions. Questions were based on two domains regarding knowledge and awareness.

Distribution of the questionnaire

The questionnaire was distributed as a Google Forms link through online platforms such as WhatsApp and email to postgraduate students, undergraduate students, and teaching faculty from various dental colleges in India. A stratified random sampling approach was used to ensure proportionate representation from each group, with participants categorized into the three aforementioned strata. Within each stratum, convenience sampling was applied to recruit participants who were accessible and willing to participate. They were allowed to complete the form only once with no time restrictions, ensuring voluntary participation. Responses were collected anonymously after obtaining informed consent and explaining the study’s aim to maintain ethical integrity.

Statistical analysis

Descriptive analysis involved presenting the explanatory and outcome variables in terms of frequency and proportions for categorical variables. In this study, Pearson's chi-squared test was used to compare the categorical responses among different groups. All statistical analyses were performed using the IBM SPSS Statistics for Windows, Version 25 (Released 2017; IBM Corp., Armonk, New York, United States). A p-value of <0.05 was considered significant.

## Results

**Figure 1:**
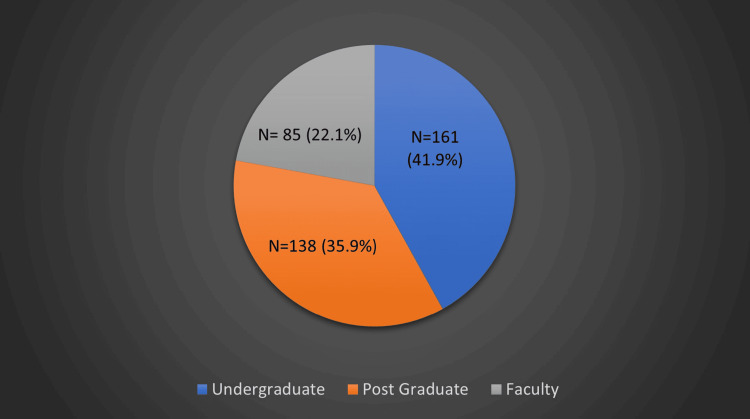
Distribution of the study participants

The study aimed to assess the knowledge, awareness, and practice of ChatGPT among dental students and members of the teaching faculty. A total of 384 participants were included, with 161 undergraduates, 138 postgraduates, and 85 members of the teaching faculty (Figure 1).the 

In the knowledge domain (Table 1), a significant majority (87.2%) knew that ChatGPT is an AI-based deep-learning neural network, with postgraduates (94.2%) demonstrating the highest level of knowledge. A similar proportion of participants (87.2%) believed that specific training is essential for crafting effective dental-related prompts for ChatGPT to ensure its optimal use in dentistry, with postgraduates (94.2%) expressing the highest need. ChatGPT's ability to guide patients to reliable resources was widely acknowledged, with postgraduates (89.9%) showing the highest level of acceptance. ChatGPT's role in assisting clinical decision-making was recognized by 56.8% of the participants overall, with the members of the teaching faculty (74.1%) showing the highest agreement, while undergraduates remained more skeptical (47.2% in favor). Most of the respondents (77.9%) agreed that ChatGPT could provide personalized preventive dental care recommendations, with the members of the teaching faculty (88.2%) being the most confident. Approximately 76.3% of the participants emphasized the importance of strict data privacy regulations, with the members of the teaching faculty (91.8%) showing the highest concern. Most of the study population (74.2%) recognized ChatGPT's potential in tele-dentistry, while 77.1% supported ChatGPT’s integration into appointment booking systems, with the members of the teaching faculty (100%) showing the highest agreement. Regarding ChatGPT’s role in generating seminar content, 82% of respondents acknowledged its potential, though this was not statistically significant (p=0.224). The ability of ChatGPT to assist in clinical decision-making was acknowledged by 56.8% of respondents overall, but undergraduate students were more skeptical, with only 47.2% agreeing with the premise.

**Table 1:** Distribution of the participant responses toward the knowledge-based questions *p<0.05 - statistically significant

Question	Response	Undergraduate N (%)	Postgraduate N (%)	Teaching faculty N (%)	Total N (%)	Chi-square	p-value
1) Do you know that ChatGPT is a type of AI-based deep learning neural network?	No	29 (18%)	8 (5.8%)	12 (14.1%)	49 (12.8)	10.141	0.001*
	Yes	132 (82%)	130 (94.2%)	73 (85.9%)	335 (87.2%)		
2) Do you think ChatGPT can help create interactive study materials?	No	29 (18%)	0 (0.0%)	12 (14.1%)	41 (10.7%)	26.63	0.001*
	Yes	132 (82%)	138 (100.0%)	73 (85.9%)	343 (89.3%)		
3) Do you know ChatGPT can generate outlines, summaries, or draft presentations?	No	33 (20.5%)	26 (18.8%)	10 (11.8%)	69 (18.0%)	2.989	0.224
	Yes	128 (79.5%)	112 (81.2%)	75 (88.2%)	315 (82.0%)		
4) Do you know ChatGPT can create and solve problem-based learning (PBL) scenarios?	No	42 (26.1%)	41 (29.7%)	7 (8.2%)	90 (23.4%)	14.603	0.001*
	Yes	119 (73.9%)	97 (70.3%)	78 (91.8%)	294 (76.6%)		
5) Do you know ChatGPT can aid in clinical decision-making?	No	85 (52.8%)	59 (42.8%)	22 (25.9%)	166 (43.2%)	16.438	0.001*
	Yes	76 (47.2%)	79 (57.2%)	63 (74.1%)	218 (56.8%)		
6) Do you know ChatGPT can direct patients to reliable online resources?	No	63 (39.1%)	14 (10.1%)	24 (28.2%)	101 (26.3%)	32.417	0.001*
	Yes	98 (60.9%)	124 (89.9%)	61 (71.8%)	283 (73.7%)		
7) Do you know ChatGPT can provide personalized preventive dental care advice?	No	50 (31.1%)	25 (18.1%)	10 (11.8%)	85 (22.1%)	14.031	0.001*
	Yes	111 (68.9%)	113 (81.9%)	75 (88.2%)	299 (77.9%)		
8) Do you know ChatGPT can be integrated into tele-dentistry for remote consultations?	No	54 (33.5%)	31 (22.5%)	14 (16.5%)	99 (25.8%)	9.71	0.008*
	Yes	107 (66.5%)	107 (77.5%)	71 (83.5%)	285 (74.2%)		
9) Do you know ChatGPT can be integrated with dental clinic appointment booking systems?	No	57 (35.4%)	31 (22.5%)	0 (0.0%)	88 (22.9%)	39.498	0.001*
	Yes	104 (64.6%)	107 (77.5%)	85 (100.0%)	296 (77.1%)		
10) Do you think AI tools like ChatGPT should ensure algorithm transparency for accountability?	No	71 (44.1%)	25 (18.1%)	21 (24.7%)	117 (30.5%)	25.392	0.001*
	Yes	90 (55.9%)	113 (81.9%)	64 (75.3%)	267 (69.5%)		
11) Should ChatGPT follow strict data privacy regulations?	No	55 (34.2%)	29 (21.0%)	7 (8.2%)	91 (23.7%)	21.537	0.001*
	Yes	106 (65.8%)	109 (79.0%)	78 (91.8%)	293 (76.3%)		
12) Should AI tools maintain high accuracy and not replace human oversight?	No	61 (37.9%)	9 (6.5%)	19 (22.4%)	89 (23.2%)	41.101	0.001*
	Yes	100 (62.1%)	129 (93.5%)	66 (77.6%)	295 (76.8%)		
13) Should AI tools support healthcare professionals rather than replace them?	No	52 (32.3%)	10 (7.2%)	0 (0.0%)	62 (16.1%)	55.464	0.001*
	Yes	109 (67.7%)	128 (92.8%)	85 (100.0%)	322 (83.9%)		
14) Do you think specific training is needed to craft effective ChatGPT dental prompts?	No	29 (18.0%)	8 (5.8%)	12 (14.1%)	49 (12.8%)	10.141	0.006*
	Yes	132 (82.0%)	130 (94.2%)	73 (85.9%)	335 (87.2%)		
15) Do you know ChatGPT can assist in gathering scientific presentation data?	No	71 (44.1%)	25 (18.1%)	21 (24.7%)	117 (30.5%)	25.392	0.001*
	Yes	90 (55.9%)	113 (81.9%)	64 (75.3%)	267 (69.5%)		

In the awareness domain (Table 2), 74.2% of the respondents overall were aware that ChatGPT can assist with writing research paper by finding relevant papers, summarizing content, polishing language, and fact-checking, with members of the teaching faculty (94.1%) showing the highest awareness, and the difference was statistically significant. A total of 83.1% of the participants reported an awareness of ChatGPT’s role in enhancing scientific presentations by providing relevant data and insights, with members of the teaching faculty (91.8%) being the most aware among the study groups. A majority of the respondents (72.7%) recognized the potential benefits of integrating ChatGPT as a supplementary educational tool for interactive learning, clinical scenario simulations, and remote learning, with postgraduates (79.7%) showing the highest level of agreement. 

**Table 2:** Distribution of the participant responses toward the awareness-based questions *p<0.05 - statistically significant

Question	Response	Undergraduate N (%)	Postgraduate N (%)	Teaching faculty N (%)	Total N (%)	Chi-square	p value
16) Are you aware that ChatGPT can aid in research paper writing (finding papers, summarizing, language polishing, fact-checking)?	No	47 (29.2%)	47 (34.1%)	5 (5.9%)	99 (25.8%)	23.509	0.001*
	Yes	114 (70.8%)	91 (65.9%)	80 (94.1%)	285 (74.2%)		
17) Are you aware that ChatGPT can gather scientific presentation data (statistics, insights, key findings)?	No	35 (21.7%)	23 (16.7%)	7 (8.2%)	65 (16.9%)	7.224	0.027*
	Yes	126 (78.3%)	115 (83.3%)	78 (91.8%)	319 (83.1%)		
18) Are you aware that integrating ChatGPT as a supplementary educational tool in dental curriculum can offer benefits (interactive learning, clinical scenario simulation, remote learning)?	No	52 (32.3%)	28 (20.3%)	25 (29.4%)	105 (27.3%)	5.628	0.06
	Yes	109 (67.7%)	110 (79.7%)	60 (70.6%)	279 (72.7%)		
19) Are you aware of ethical issues while using ChatGPT in healthcare?	No	58 (36.0%)	19 (13.8%)	13 (15.3%)	90 (23.4%)	24.547	0.000*
	Yes	103 (64.0%)	119 (86.2%)	72 (84.7%)	294 (76.6%)		

Within the practice domain (Table 3), when queried about using ChatGPT as a supplement to prepare presentations or talks, postgraduates (60.1%) were the most likely to use it. Members of the teaching faculty were the least likely to use ChatGPT for this purpose (32.9%), showing a significant difference between the groups (p=0.000). Postgraduates (81.2%) were the highest users of ChatGPT for gaining additional information about dental health topics, followed by undergraduates (53.4%). Regarding the use of ChatGPT as a knowledge repository for dentists, postgraduates (74.6%) were the most frequent users, followed by the undergraduates and then the teaching faculty.

**Table 3:** Distribution of the participant responses toward practice-based questions *p<0.05 - statistically significant

Question	Response	Undergraduate N (%)	Postgraduate N (%)	Teaching faculty N (%)	Total N (%)	Chi-square	p value
20) Have you used ChatGPT as a knowledge repository for dentists (access to updated information, research papers, guidelines for continuing education)?	No	75 (46.6%)	35 (25.4%)	47 (55.3%)	157 (40.9%)	23.223	0.000*
	Yes	86 (53.4%)	103 (74.6%)	38 (44.7%)	227 (59.1%)		
21) Have you used ChatGPT as a supplement to prepare your presentations or talks?	No	72 (44.7%)	55 (39.9%)	57 (67.1%)	184 (47.9%)	16.733	0.000*
	Yes	89 (55.3%)	83 (60.1%)	28 (32.9%)	200 (52.1%)		
22) Have you used ChatGPT to gain additional information regarding any dental health problems or topics?	No	75 (46.6%)	26 (18.8%)	45 (52.9%)	146 (38.0%)	34.583	0.000*
	Yes	86 (53.4%)	112 (81.2%)	40 (47.1%)	238 (62.0%)		

## Discussion

AI tools, such as ChatGPT, have emerged as invaluable assets in the field of dentistry, significantly enhancing the speed and quality of content creation. Developed by OpenAI, ChatGPT leverages advanced natural language processing (NLP) techniques to produce text that is not only contextually relevant but also medically accurate [10]. This capability makes it an essential resource for dental professionals, educators, and researchers who seek to stay informed and share knowledge effectively. The rapid advancement of AI technologies holds great promise for various aspects of dentistry, including diagnosis, treatment, and prognosis [11]. This integration of AI is revolutionizing the field by fostering greater precision in procedures, decreasing the likelihood of errors, and reducing the dependency on humans. In dental clinics, AI can take on an array of practical tasks that streamline operations. For instance, it can efficiently schedule patient appointments, manage follow-ups, and assist dental practitioners in clinical diagnosis and treatment planning [12]. By automating these processes, AI not only enhances the overall patient experience but also allows dental teams to focus more on delivering high-quality care [13].

The results of this study reveal a clear trend in the knowledge, awareness, and usage practices of ChatGPT across different educational groups. Overall, postgraduate students exhibited a significantly higher level of understanding and use of ChatGPT compared to undergraduate students and members of the teaching faculty. This could be attributed to their advanced academic exposure and the more frequent interaction with technological tools in their research and clinical activities. This is in line with the increasing integration of AI tools into advanced educational settings. The significant differences observed across groups in the areas of problem-based learning (PBL), clinical decision-making, and patient management highlight a growing recognition of ChatGPT’s potential in enhancing educational and clinical workflows.

Members of the teaching faculty, with their experience in clinical practice and teaching, demonstrated a greater awareness of the role AI can play in these areas. This suggests that they are more attuned to the practical applications of such technologies, especially in guiding students through real-life clinical scenarios. The teaching faculty and the postgraduate students showed greater awareness of ChatGPT’s role in research paper writing compared to undergraduates. This supports the findings of Belaldavar et al. wherein more than 89% of participants believed ChatGPT can assist with tasks like data collection, literature reviews, and writing manuscripts [14]. Interestingly, although there were significant differences in awareness levels across the groups, the general consensus leaned toward using ChatGPT as a supportive tool rather than a replacement for human expertise. This reflects a broader concern in the healthcare sector about maintaining human oversight in decision-making, particularly in clinical settings. The emphasis on accuracy and the ethical use of AI further strengthens this viewpoint, suggesting that while AI tools like ChatGPT can greatly enhance healthcare processes, they must be employed responsibly, with careful consideration of privacy, security, and professional ethics. The teaching faculty, the undergraduates, and the postgraduates recognized the potential benefits of integrating ChatGPT into the dental curriculum, particularly for interactive learning and remote education. This aligns with the findings of Shete et al., who emphasized the need for dental educators to adapt teaching methods to enhance student learning [15]. Despite these positive trends, the findings also indicate that undergraduate students, who make up the largest group in this study, have comparatively lower levels of awareness and usage of ChatGPT. This highlights the need for more widespread education and integration of AI technologies into the undergraduate dental curriculum. The moderate awareness of integrating ChatGPT into the dental curriculum suggests that educational institutions may have not fully exploited the potential of AI tools for early-stage dental education. This presents an opportunity for curriculum developers to incorporate more AI-focused modules, ensuring that future dental professionals are well-versed in the use of these technologies.

Since the study relied on a self-reported questionnaire, there is a potential for self-report bias, where participants may have overestimated or underestimated their familiarity with and use of AI tools. Misinterpretation of AI-related questions is another possible limitation, as varying levels of exposure to AI technologies might have led to differences in how participants understood and responded to survey items. Furthermore, the sampling approach may have introduced selection bias, as participants who were already interested in AI might have been more likely to participate in the study, potentially skewing the findings toward a more favorable view of AI in dentistry. Future research should address these limitations by incorporating a larger and more diverse sample across different regions of India and refining the design of the questionnaire to reduce response bias and enhance accuracy in assessing AI knowledge and usage.

## Conclusions

Considering the limitations of this study, the findings suggest that postgraduate students exhibit higher levels of awareness and engagement with AI tools like ChatGPT in dentistry than undergraduate students and members of the teaching faculty. However, there remains a need for increased exposure to and training in AI for undergraduate students and faculty to enhance their understanding and ensure the ethical use of these technologies. The teaching faculty must develop proficiency in AI to effectively guide both postgraduate and undergraduate students in its appropriate application. While AI holds potential in modern dental education and practice, this study primarily assesses knowledge and awareness rather than its direct impact. Therefore, further research is needed to evaluate how AI integration influences actual learning outcomes, clinical decision-making, and patient care. As AI continues to develop, structured training programs focusing on crafting precise and contextually relevant dental-related prompts could help optimize its use. By fostering a well-informed approach to AI in dental education, institutions can better prepare future dental professionals to engage with these evolving technologies responsibly and effectively.
